# Simple yet effective: Historical proximity variables improve the species distribution models for invasive giant hogweed (*Heracleum mantegazzianum* s.l.) in Poland

**DOI:** 10.1371/journal.pone.0184677

**Published:** 2017-09-19

**Authors:** Piotr Mędrzycki, Ingeborga Jarzyna, Artur Obidziński, Barbara Tokarska-Guzik, Zofia Sotek, Piotr Pabjanek, Adam Pytlarczyk, Izabela Sachajdakiewicz

**Affiliations:** 1 Laboratory of Applied Plant Ecology, Faculty of Ecology, University of Ecology and Management in Warsaw, Warsaw, Poland; 2 Department of Plant Ecology and Environmental Protection, Faculty of Biology, Biological and Chemical Research Centre, University of Warsaw, Warsaw, Poland; 3 Department of Forest Botany, Faculty of Forestry, Warsaw University of Life Sciences, Warsaw, Poland; 4 Deptartment of Botany and Nature Protection, Faculty of Biology and Environmental Protection, University of Silesia in Katowice, Katowice, Poland; 5 Department of Botany and Nature Conservation, Faculty of Biology, University of Szczecin, Szczecin, Poland; 6 Department of Geoinformatics, Cartography and Remote Sensing, Faculty of Geography and Regional Studies, University of Warsaw, Warsaw, Poland; Universita degli Studi di Napoli Federico II, ITALY

## Abstract

Species distribution models are scarcely applicable to invasive species because of their breaking of the models’ assumptions. So far, few mechanistic, semi-mechanistic or statistical solutions like dispersal constraints or propagule limitation have been applied. We evaluated a novel quasi-semi-mechanistic approach for regional scale models, using historical proximity variables (HPV) representing a state of the population in a given moment in the past. Our aim was to test the effects of addition of HPV sets of different minimal recentness, information capacity and the total number of variables on the quality of the species distribution model for *Heracleum mantegazzianum* on 116000 km^2^ in Poland. As environmental predictors, we used fragments of 103 1×1 km, world- wide, free-access rasters from WorldGrids.org. Single and ensemble models were computed using BIOMOD2 package 3.1.47 working in R environment 3.1.0. The addition of HPV improved the quality of single and ensemble models from poor to good and excellent. The quality was the highest for the variants with HPVs based on the distance from the most recent past occurrences. It was mostly affected by the algorithm type, but all HPV traits (minimal recentness, information capacity, model type or the number of the time periods) were significantly important determinants. The addition of HPVs improved the quality of current projections, raising the occurrence probability in regions where the species had occurred before. We conclude that HPV addition enables semi-realistic estimation of the rate of spread and can be applied to the short-term forecasting of invasive or declining species, which also break equal-dispersal probability assumptions.

## Introduction

Species Distribution Models (SDMs) are widely used in nature conservation and management practice. In recent years they have been applied to unstable invasive species (*invasive species distribution models—*iSDMs) [1,2]. In order to be reliable for modelling of any species, SDMs have to meet many assumptions. The most important, called the *equilibrium assumption* [3], states that a modelled species must be in an equilibrium with the area of interest, i.e. it should have an equal probability of occurrence in every point inside the geographical space or in a set of all unique combinations of different states of predictor variables, i.e. in environmental hyper-space [4]. Depending on the stage of invasion, invasive species (IS) more or less fail to meet this assumption [5,6]. In the initial phase starting just after passing large geographical barriers, IS are usually extremely rare and occur in a few–more or less random–point, line or small polygon localisations and thus do not occur in all places that are susceptible to invasion [7–10]. In the next–space infilling–stage they disperse from initial places. Depending on the availability of the suitable habitats, and often connected to human land use [11,12] as well as the efficiency of short- and long-distance dispersal, it may result in different levels of patchiness in distribution, especially when mechanisms responsible for near- and long-distance dispersal are separate [13–14]

As in every diffusion process, the patchy spatial pattern is strongly, positively autocorrelated, because the local density at any place is a function of previous densities at this and neighbouring places [15]. This results in spatial autocorrelation (SAC) which causes problems in spatial statistical inferring, including iSDMs.

**Fig 1:**
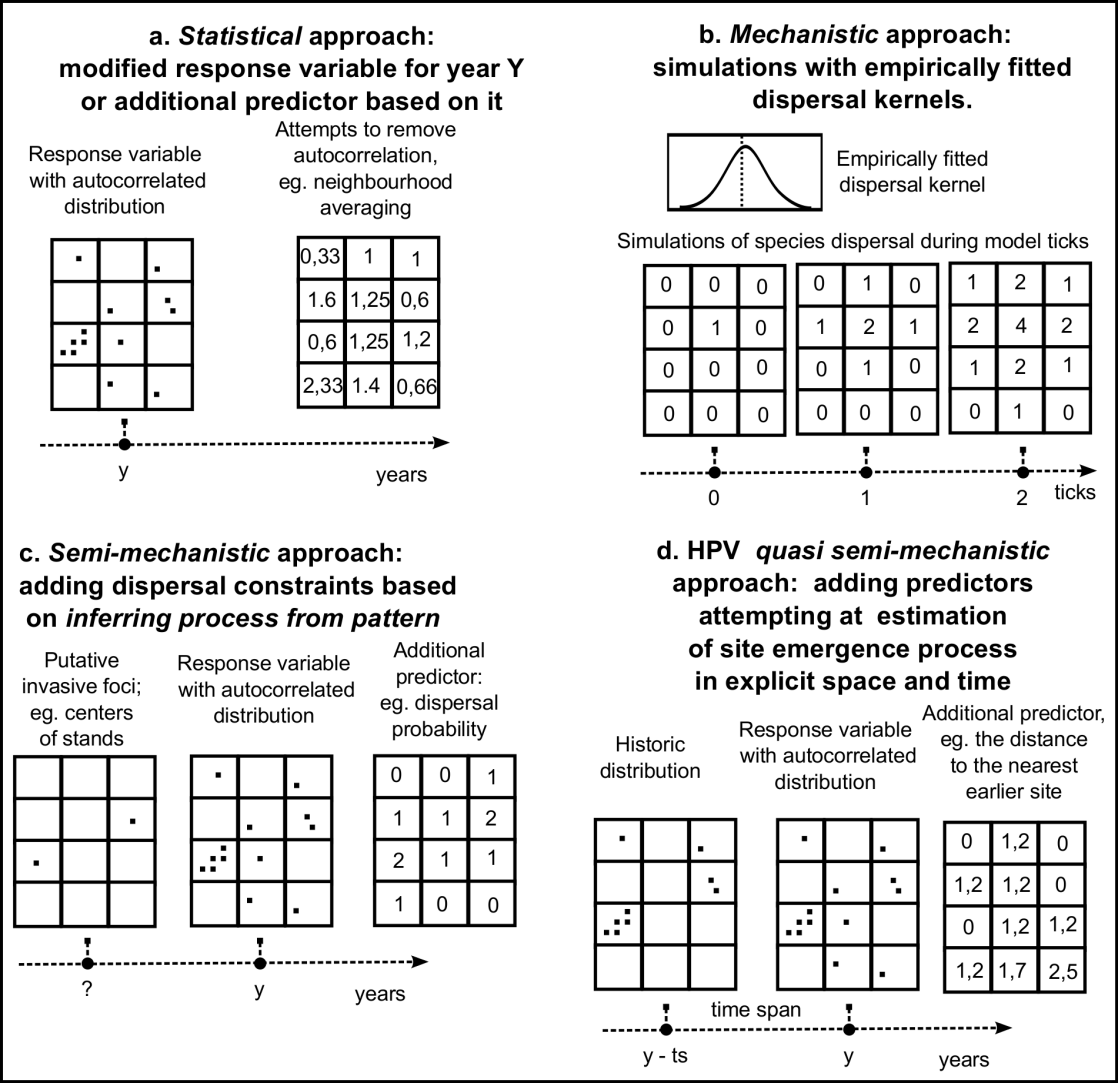
The comparison of the historical proximity-based quasi semi-mechanistic approach to modelling of the invasive plant species with SAC in their distribution with existing statistical, mechanistic and semi-mechanistic approaches.

The usual statistical approach involves calculating additional predictors representing the intensity of the invasion phenomenon in the vicinity of a given model cell, e.g. the neighbourhood averaging or sum of abundance or the count of sites in the direct neighbourhood apart from the cell itself. The predictor may be also autocovariate in an autologistic model (Fig 1A). Various mechanistic approaches attempt to recreate the web of causal relationships that produce the distribution with SAC. Usually it requires previous empirical fitting of the model parameters, like the distributions of the distance of dispersal, expressed as dispersal kernel (Fig 1B). A semi-statistical approach uses additional predictors, e.g. probability of propagule immigration from the alleged initial sites into each model cell, computed from the predictor data (Fig 1C). SDMs based on historical proximity variables (HPV) extend this semi-mechanistic approach by adding to SDM one or more predictors, representing, e.g. the distance to the nearest site existing in a precisely set moment in the past (Fig 1D).

Many remedies have been proposed in order to compensate for the consequences of assumption breakage in autocorrelated data analysis. SAC in non-spatial statistics for bivariate data, like Pearson's correlation, can be compensated for by decreasing the number of degrees of freedom, which is considered to remove information redundancy resulting from SAC [3,16]. However, these simple solutions are not applicable to all SAC problems, e.g. they are not suitable for a case where distance to independence cannot be estimated and the spatial dependence may not be defined as simple function of a distance.

In spatial statistics, including SDMs, equilibrium assumption breakage due to SAC has been accounted for either through statistical or mechanistic approach. In the statistical approach, spatial corrections to predictions are used [3]. In autologistic models the standard set of environmental predictors is extended by an additional variable, called the autocovariate (Fig 1A), which accounts for the correlation between the value of the response variable at the location and in its neighbourhood [17]. Other methods, like spatial eigenvector mapping, generalised least squares and generalised estimating equations are also available [18].

**Statistical approach** models have the ability to explain large-scale distribution patterns, from regional to continental scales using climatic or topographic indices or soil maps. They are also relatively easy to implement, because they do not require expert knowledge or empirical data for model calibration [19]. However their outcome is static, and they often lack the ability to predict future or potential distributions of invasive species. They also ignore or mask the causal relationships behind the autocorrelated spatial pattern [3].

**Mechanistic approach** models allow for simulation of the process, but are either too simple and unrealistic or, when more complex, require labour-intensive parametrisation [4,20,21]. In mechanistic models of invasions, one tries to simulate the effects of invasion-process constraints linked with habitat, dispersal patterns or propagule pressure [14]. The most common form of limitation is the dispersal limitation, which can be simulated in many different ways, e.g. dispersal constraints [1] or dispersal kernels, i.e. the distribution of probabilities of species immigration at any given distance from the source (Fig 1B). A few recent models, like the recent R-based MIGCLIM model [22,23], allow for an integration of cellular automata with dispersal constraints onto habitat suitability models produced, e.g. by the BIOMOD package [24,25]. However, even the MIGCLIM package requires species-specific parameters to simulate its dispersal. Therefore, the empirical calibration of dispersal is still required.

**Semi-mechanistic approach** models are intended to incorporate most advantages of both approaches without inheriting their weaknesses [21,26]. Instead of empirical fitting and simulational verification of dispersal kernels it attempts to *infer process from pattern* (Fig 1C) using the highly effective regressive ability of SDM models [19,27]. The best examples of this attempt are models of *Accacia saligna*, *A*. *cyclops* and *Pinus pinaster* in Cape Province [28]. In this study, the probability of dispersal was estimated through adding to the SDM model an additional predictor which was the distance to the putative initial foci of invasive spread. Such models were semi-mechanistic models, because they did not recreate the mechanism for invasion course over time but nevertheless managed to estimate and include the effect of dispersal on observed, highly autocorrelated spatial distributions in explaining and predicting. While semi-mechanistic “inferring process from pattern” approach is still not common in modern SDMs, it is suggested as a promising future solution [19].

We tested the model quality of an HPV approach in comparison with the bare SDM as a control model. We used *Heracleum mantegazzianum* s.l.*–*giant hogweed–as a test plant. It is a joint taxonomical unit, including *H*. *mantegazzianum* Sommier et Levier and *H*. *sosnowskyi* Manden [29]. Since the end of 19^th^ Century, this Caucasian perennial has become invasive in many parts of the Northern Hemisphere, including North America and Northern, Central and Eastern Europe [30–33]. In many parts of the new range it has non-even spatial distribution, either in continental, regional or local spatial scales. For example, it is much more widespread in Great Britain and southern Scandinavia than in France [30]. The fast increase of the number of sites has been observed in many regions in non-native range, e.g. in the Czech Republic [34,35].

There have been many attempts at modelling the distribution of giant hogweed using either *statistical* or *mechanistic* approaches. E.g. *autologistic models* were applied to the 10×10 km octade data for the national scale and 2×2 km tetrade data for regional scale in Great Britain [34]. It was also applied to the data on giant hogweed occurrence in Denmark at both national and regional scales [36]. The GLMM models were implemented for the local abundance of giant hogweed in 1×1 km study areas in Germany [37]. The estimation of the continent-scale potential range of giant hogweed has been done using the BIOCLIM algorithm and Global Biodiversity Information Facility data [8]. Maxent and BIOMOD ensemble SDM models were applied for *Heracleum sosnowskyi* in the Ukrainian Carpathians [33,38]. The mechanistic approach was implemented in models of the abundance of giant hogweed in polygonal, homogenous landscape patches inside 1×1 km study areas in Germany [39]. In a set of studies, spatially explicit mechanistic individually based models were applied to the demography of individuals inside a single landscape patch [40–42].

The aim of this study is to test how the addition of Historical Proximity Variables (HPV) quantifying distance to the nearest occurrence sites in different time periods in the past affects the quality of the Species Distribution Model (SDM) of a current distribution of a spreading, invasive giant hogweed in Central and Northern Poland (116,000 km^2^). HPVs tested in the article differed in: minimal recentness (25, 10 and 5 yrs earlier than response data), information capacity (which is low for a variable describing the simple presence or absence of previous sites; moderate, in the case of the count of previous sites; and high, in the case of distance to the nearest previously existing site) and the total number of HPVs included in a dataset. We tested effects of the addition of one or more HPV on the model quality True Skill Statistic (TSS) of giant hogweed.

In this paper we propose and evaluate an improvement for iSDMs in which we account for SAC in regional-scale iSDM modelling of an invasive plant species with patchy distribution during an infilling phase (Fig 1D). In comparison to the typical semi-mechanistic approach, there is no separate mechanical model subsystem; however, the spatial dependence resulting from the dispersal process is emulated by inclusion of one or more historical proximity variables (HPV), allowing the SDM to estimate the possible spatial relation between the current and past distribution in earlier stages of invasion. HPV predictors are based on the spatial distributions from explicitly defined time periods (5–25 years) before the date that the response variable distribution was recorded. In order for such a model to be good enough, it should improve an explanation, current distribution projections, or projections of the future expanded range, perhaps even as a time series. The proper estimation of the relative importance of the dispersal from the earlier sites should allow for better projection of potential distribution of an invasive species, using an assumption of uniform, little or no dispersal constraint.

We expected that:

1. an addition of Historical Proximity Variables would increase model quality in comparison with exclusively environmental Spatial Distribution Models, both in single and ensemble models;
2. the number of time periods, minimal recentness, information capacity and the type of algorithm used would all significantly influence the increase in model quality; and
3. the improved models would be an interesting method for performing an SDM-based short-term forecast for invasive species and other spatially unsaturated taxa.

## Materials and methods

### Giant hogweed distribution data

**Fig 2:**
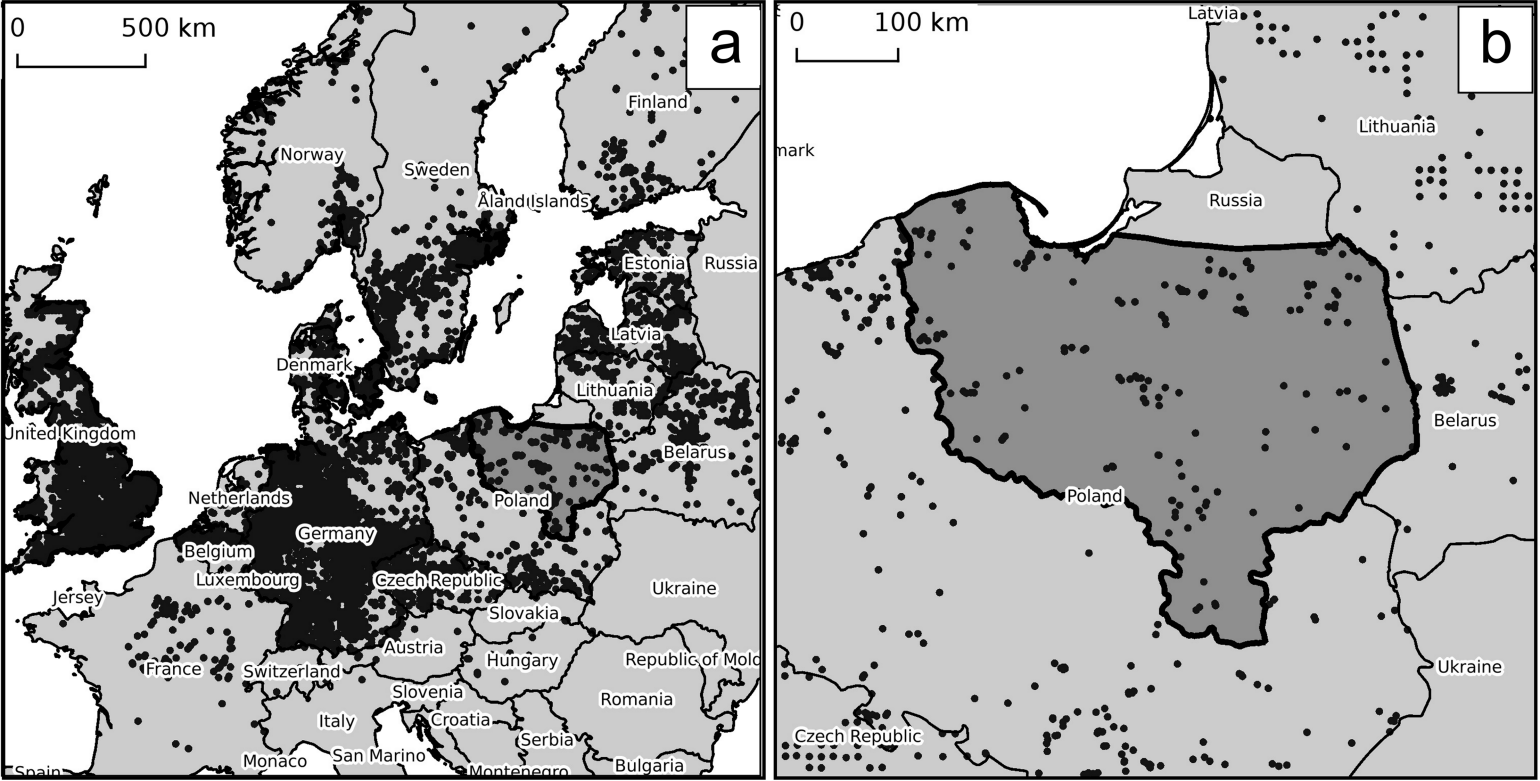
**The localisation of the study area within the range of giant hogweed in (a) Europe and (b) in Poland**. Distribution map–the data from Global Biodiversity Information Facility: [43], except for Poland [44], Belarus [44–46], Lithuania, Latvia and Estonia [30]. The study area is indicated by a dark grey colour.

The data for giant hogweed we used (Fig 2) come from the time period of the initial colonisation and space infilling stage [33]. In some countries, e.g. in Great Britain or in Germany, giant hogweed has a relatively dense and even distribution (Fig 2A), while in other countries it has a sparse distribution with many new, recent sites, e.g. in Latvia, Estonia, Belarus and Poland. The population of giant hogweed in Poland (Fig 2B) has increased from 196 sites presented as 146 ATPOL Atlas squares (10×10 km) in 2001 [43] to ca. 500 in 2011 and 1710 in 2014 [44].

As a response variable we used data collected in 2012 for the territory of Poland during a civic science project [47]. During that project, data were collected from heterogeneous sources between May and November 2012 from 673 national, regional and local institutions, as well as municipalities located at least 50 km from previously known giant hogweed sites. Personal observations were also gathered through the project’s website (http://barszcz.edu.pl), Facebook fan page and e-mail. If reported giant hogweed localities were different than those previously noted, a confirmation was attempted by direct contact with the authors of the record, and the maximum possible examination of evidence (photos, videos or detailed descriptions of observed individuals) was performed. In total, 1710 sites were identified in the whole country, out of which 628 were located within the research area. The total number of data points was further amended by 1000 pseudoabsences, located randomly within the reserch area. Therefore the total number of analysed cases was 1628.

### Historical proximity variables

**Fig 3:**
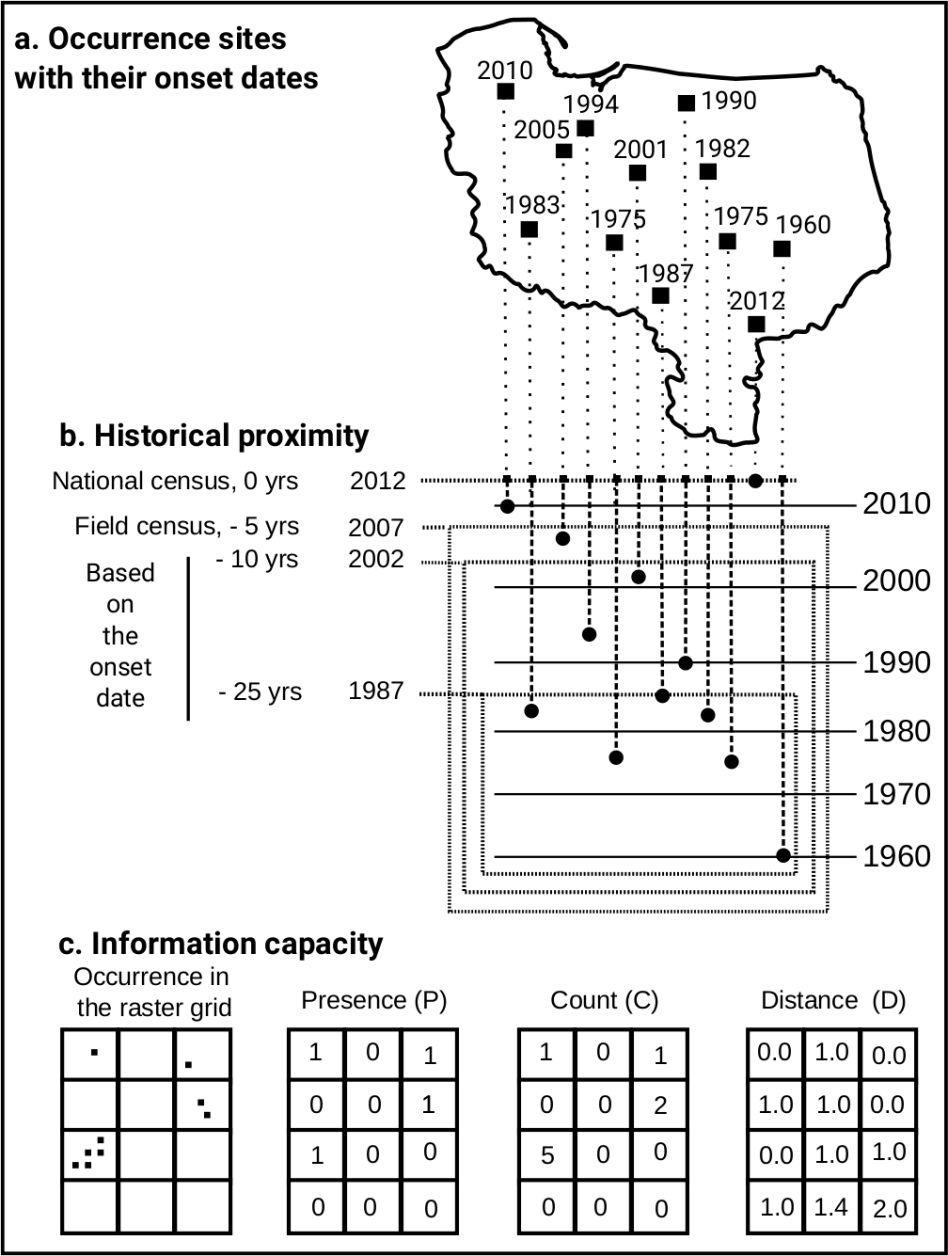
Generation process of historical proximity variables.

Data on the historical existence of giant hogweed sites were taken from the field census of giant hogweed populations, performed in May-June 2007 in Central and Northeastern Poland over 116,000 km^2^ (Fig 3A and 3B, [48]). In this census giant hogweed sites found in both peer-reviewed and grey literature were verified. If found, the geographical location of the center and the spatial extension of the local population were noted with a Garmin E-Trex H GPS unit within an accuracy of minimum 10 m. Populations distant more than 500 m one from another were treated as separate ones. Any new locations encountered during the census were mapped in the same way. During the 2007 field census, in total 101 localities were eventually found.

Earlier history (before 2007) was examined by setting a population’s onset date (Fig 3A), defined as the earliest year at which the given giant hogweed population at the given locality had been known, either described in published sources or remembered by eyewitnesses. If the exact date was not available, decennial estimation was attributed, in the form of the middle year of the decade (e.g. 1975 for the years 1970–1979).

Based on these data, three partially overlapping subsets were defined (Fig 3B):

- all localities existing 5 years before the 2012 census (all present in 2007),
- all localities existing 10 years before the 2012 census (onset date < = 2002),
- all localities existing 25 years before the 2012 census (onset year < = 1987).

Data from the census were projected onto the http://worldgrids.org Level3 geographical grid with cell size of 1/120 degree, i.e. ~1×1 km [47] using the *rasterize()* function from the R package raster 2.2–31 with the count of locations as a grouping function. Three different levels of information capacity were then extracted (Fig 3C):

- ‘Presence’ of sites inside a single raster cell, as a conversion of count into presence-absence format (expressed as “P” at the end of set labels);
- ‘Count’ as a number of sites inside the raster cell, a direct result of the *rasterize()* function from the package *raster* (labeled as “C”);
- ‘Distance’, as a distance between each raster cell and the nearest occurrence site, using the raster package’s *distanceFromPoints()* function with default settings (labeled as “D”).

If both ‘Count’ and ‘Distance’ were applied, the “CD” index was used.

### Historical proximity variables variants

Each kind of HPV was computed for each time period. Thus 24 combinations of the standard set of general variables exist–with the HPVs added, named after the HPV name and time period: e.g. “5.P” is a variant with presence data from the time period of “5 years before”, and “25.10.5.CD” is a variant with both Count and Distance from the time periods of “25 years before”, “10 years before” and “5 years before”. There was one control variant (“No HPV”), with no historical proximity variables added.

### Environmental explanatory variables

As environmental predictors, we used the world-wide, free-access rasters prepared for the World Soil Map made available by ISRIC—World Data Centre for Soils, from the WorldGrids website [47]. We applied 105 Level3 geographical grids (1/120 degree, i.e. ~1×1 km). The data comprising 90 categories belonged to nine groups: administrative and socio-economic, climatic and meteorological, morphometric, land cover and land use, bioclimatic, urbanization and lights at night, geological and soil parent material, natural hazard, and forest and wildlife data (S1 Table). Their metadata with detailed description are available from the WorldGrids.org website (http://worldgrids.org/doku.php?id=wiki:layers) [49]. The time coverage of the data is similar to that of the modelled phenomena (~1987–2012). A few variables used in the analysis were rescaled to the size of the used grid. Global predictors were cropped to the extent of the study area. Some variables that were available only as Level1 grids (mainly soil data from the Harmonized World Soil Database) were scaled up to the Level3 degree resolution. All rasters with no variation inside the study area were omitted.

The authors are aware that the limited spatio-temporal correlation of the occurrence and abundance data with predictor values may weaken the detection ability of less strong spatial relationships. Data on the onset date of localities are not perfect, as there was no monitoring at that time. There is a possibility that onset dates of some localities should be earlier than was established or that more unknown sites existed before 2011, but there are no better historical data so far. The model quality data and variables’ importance should be considered conservative estimations.

### Statistical analysis

The modelling framework used in this study was BIOMOD2 package 3.1.47 working in R environment 3.1.0. [50,51] with spatial point data as a response variable and the series of stack of raster layers as predictors. We chose 7 of 10 available algorithms (Artificial Neural Networks–ANN, Classification Tree Analysis–CTA, Flexible Discrimination Analysis–FDA, Gradient Boosting Machines–GBM, Multiple Adaptive Regression Splines–MARS, Random Forests–RF and Surface Range Enveloppe–SRE), that were fastest and the most flexible. We omitted algorithms with restrictive error distribution requirements (like Generalized Linear Models–GLM and Generalized Additive Models–GAM) or posing technical problems (Maximum Entropy models–MAXENT).

In order to assess the effect of an algorithm and HPV characteristics on the model quality, we melted modelling results using the function *melt()* from the *reshape2* package, row-binded using the *rbind()* function, and then added the following HPV set and model attributes:

- model algorithm used,
- combination of information capacity variables (“P”, “C”, “D”, “CD” or control),
- minimal recentness of time periods included (5, 10, 25 years or control),
- number of time periods included (1, 2, 3 or control).

Graphical analysis was performed using box plots with default settings from the *latticist* package.

True skill statistic (TSS) was used as a measure of the model quality, since it was a default measure in BIOMOD 2.1.37 [52]. TSS values below 0.4 are considered moderate; 0.4–0.6 are good [53]. In BIOMOD2 only single models with TSS ≥ 0.7 were considered good enough to be chosen for ensemble models [25]. The second default model quality measure in BIOMOD2 software is ROC. Both TSS and ROC are often used in modelling papers. Both are robust, prevalence- independent methods. Their values differ slightly, with TSS being somewhat more variable, i.e. giving relatively lower scores for weaker models. However, the differences between their values are rather a matter for statistical debate [54–58]

In order to assess the importance and significance of HPV properties we performed a meta-modelling, using melted BIOMOD2 modelling results as a dataset. As a meta-model algorithm we used a *Boruta()* function from the Boruta 3.1 package [59]. It uses a large number of *randomForest()* function model iterations and classifies all variables into three categories: ‘Confirmed’, ‘Tentative’ and ‘Rejected’, based on the significance test comparing the mean value of the Z-score for all variables with Z-scores of their randomized copies. ‘Confirmed’ status was attributed to variables whose Z-score differed significantly at p = 0.01, which means that they were significantly important for model quality. TSS values for single and ensemble models were standardized inside these groups.

## Results

### Quality of models with and without historical proximity variables

**Fig 4:**
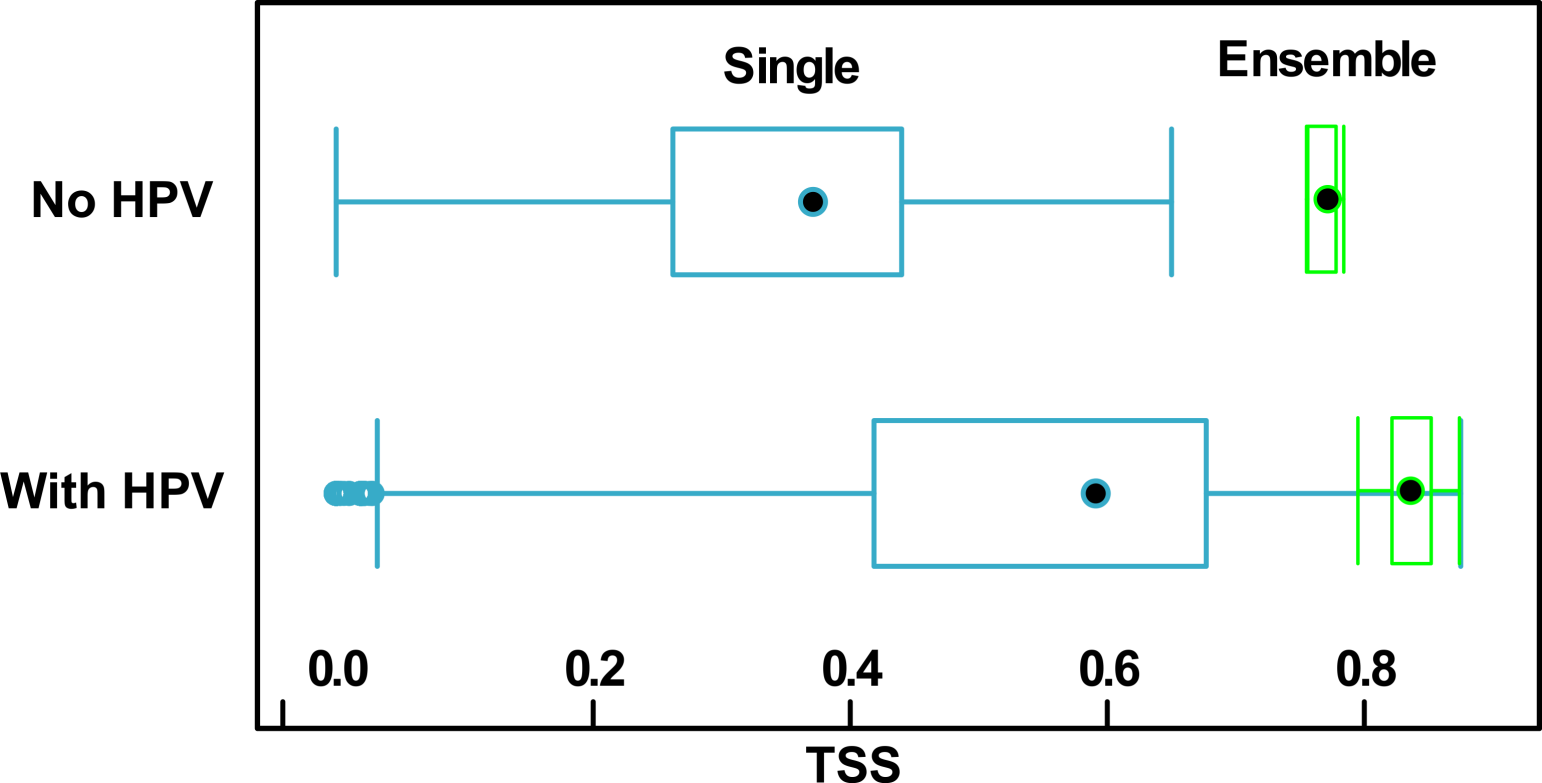
TSS quality values of single (blue colour) and ensemble (green colour) BIOMOD2 models for variants with and without historical proximity variables (HPV).

Models with HPV scored better than the control variant with environmental predictors only (Fig 4). The average quality of single models of the control variant was poor. They had a median TSS value of 0.38 and InterQuartileRange (IQR) extending from 0.26 to 0.44. The ensemble models for this variant were based on very few models that passed a 0.7 threshold. Their average TSS value was 0.77, and IQR ranged from 0.74 to 0.79. The average quality of single models with HPV was at least good, i.e. with median TSS = 0.6 and IQR ranging from 0.42 to 0.68, while the median TSS value of ensemble models with HPV was an excellent 0.82, with IQR from 0.8 to 0.85.

### The comparison of historical proximity variables variants

**Fig 5:**
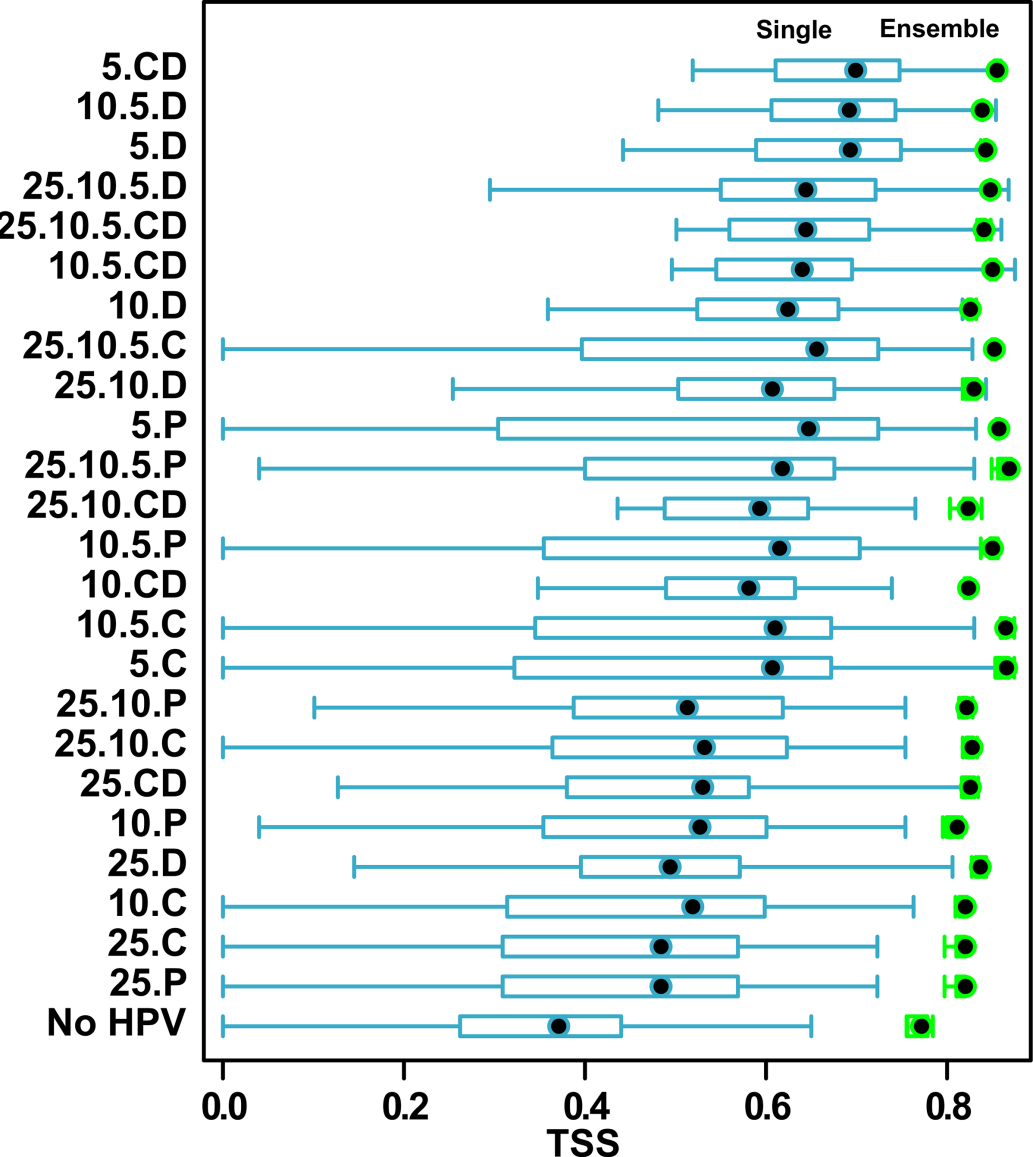
TSS quality values for single (blue) and ensemble (green) BIOMOD2 models produced using different historical proximity variables (HPV) variants. Types of data included: P–presence, C–count, D–distance; 5, 10, 25 –minimal recentness predictors expressed in number of years; detailed explanation in the text.

Not all HPV sets performed equally well (Fig 5). The median TSS value was the highest (0.68) for the most recent ‘Distance predictors (5.CD, 10.5.D and 5.D) and the lowest (0.48) were for variants least recent predictors of all kinds: ‘Distance’, ‘Count’ and ‘Presence’ (25.D, 25.C and 25.P). The control variant with no HPVs had the lowest quality (0.38). The variation of TSS values for ensemble models was much smaller, regardless of the HPV variant. The variants with the highest median TSS values were those with HPV from the most recent time period–mainly based on 'Distance' (5.CD, 10.5.D, 5.D, 25.10.5D, 25.10.5CD, 10D).

### The role of historical proximity variables set attributes

**Fig 6:**
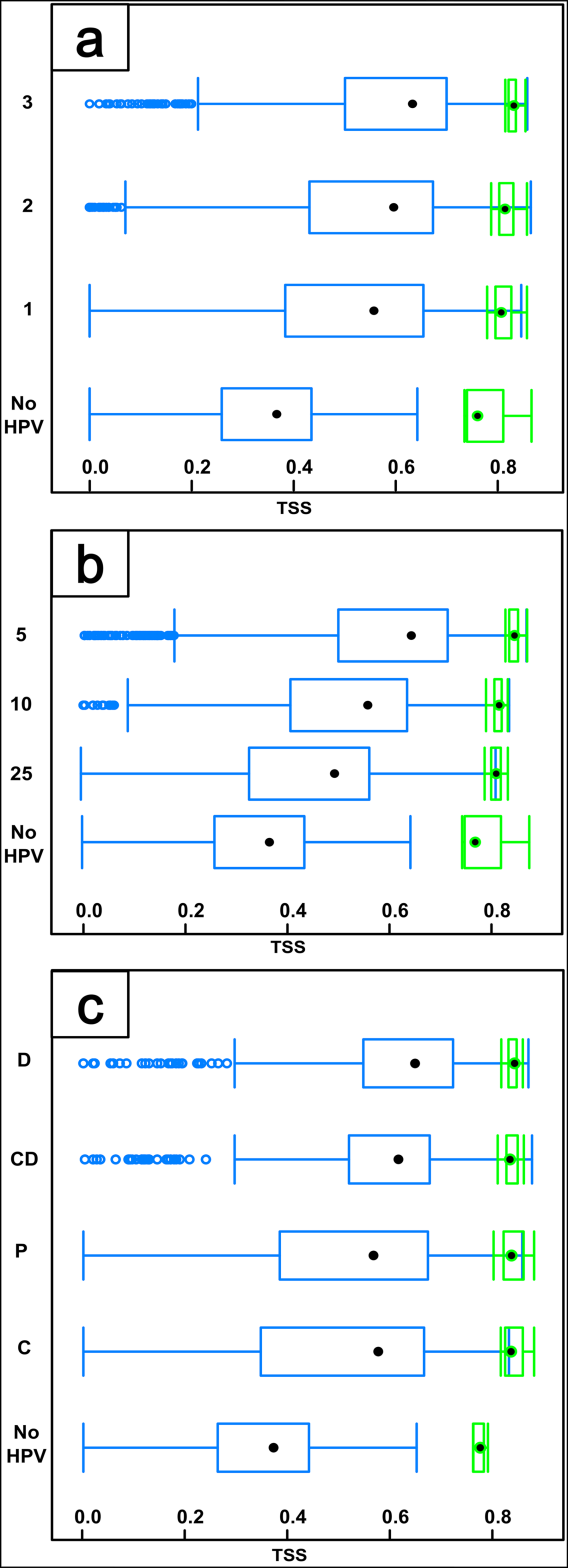
TSS quality values for single (blue) and ensemble (green) BIOMOD2 models produced using historical proximity variables (HPV) sets differing in the number of time periods (a), minimal recentness (b) and information capacity of HPVs (c).

HPV set attributes affect TSS values in different ways (Fig 6). Model quality expressed by the median TSS value increases by 0.05 between consecutive numbers of time periods (Fig 6A). This increase is one third the amount of the difference between sets with one time period (TSS = 0.52) and the control (TSS = 0.37, median TSS difference = 0.15).

Model quality is clearly affected by HPV set minimal recentness (Fig 6B). TSS median value drops by 0.1 when increasing minimal recentness categories from 5 to 25. Information capacity affects model quality less than minimal recentness (Fig 6C). Models for HPV sets with ‘Distance’ proximity variables were only slightly better than ‘Distance and Count’ HPV sets (~0.05 TSS difference), which performed better than models for sets with ‘Count’ alone or ‘Presence’ (~0.1 TSS difference). Variation of TSS median values for ensemble models is similar to that for single models, but the range of values is much smaller (0.03).

**Fig 7:**
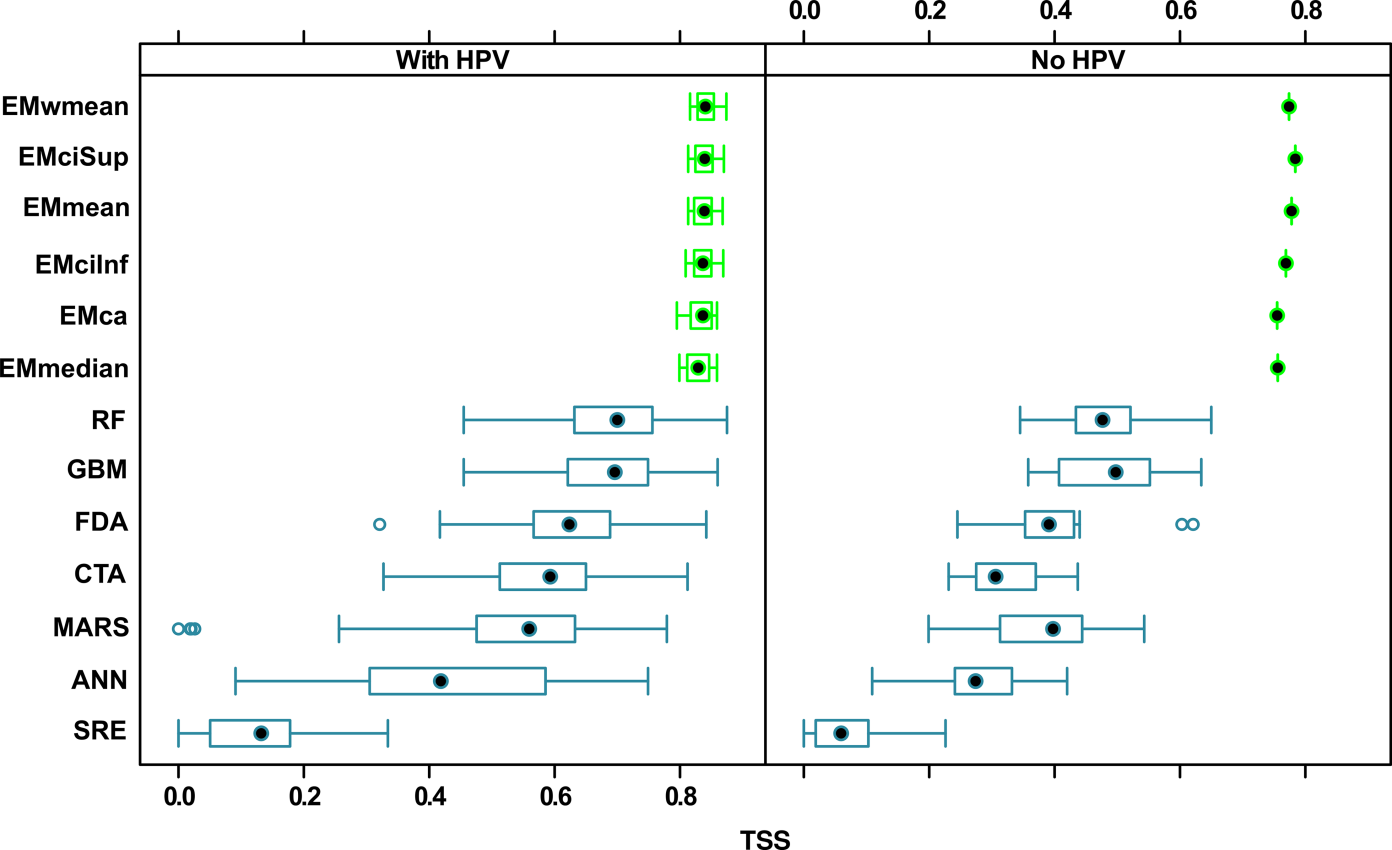
TSS quality values for single (blue) and ensemble (green) BIOMOD2 models produced using different algorithms, with and without historical proximity variables (HPV). EMmean—Ensemble Model mean; EMwmean—Ensemble Model weighted mean; EMciSup—Ensemble Model confidence interval Superior values; EMcilnf—Ensemble Model confidence interval Inferior values; EMca—Ensemble Model community averaging; EMmedian—Ensemble Model median; RF- RandomForest; GMB—Gradien Boosted Machines; FDA—Flexible Discrimination Analysis; CTA—Classification Tree Analysis; MARS—Multiple Adaptive Regression Splines; ANN—Artificial Neural Networks; SRE—Surface Range Envelope.

Regardless of the algorithm used, the median TSS values of ensemble models were much higher than those of single models (Fig 7), both for sets with HPV (0.81–0.83 vs 0.14–0.69) and without HPV (0.76–0.79 vs 0.16–0.51). Single model algorithms diverged into three groups. RF and GBM gave the best prediction (median TSS = ~0.7 for sets with HPV and ~0.5 for sets without HPV). FDA, MARS, CTA and ANN model quality was lower (median TSS from 0.4 to 0.6 for sets with HPV and from 0.28 to 0.4 for sets without HPV). SRE was the worst model (median TSS = 0.13 for sets with HPV and 0.06 for sets without HPV). Single models, especially ANN, had much higher level of variation in TSS values than ensemble models. All ensemble models worked almost perfectly, and weighted mean was the best ensemble model algorithm.

### The importance and significance of historical proximity variables set attributes

All tested HPV set attributes were classified as 'Confirmed' by the Boruta meta-model, i.e. each one of them is a significantly important predictor of the model quality at p ≤ 0.01 (Table 1). The most important determinant of the model quality was the kind of the algorithm both for the simple and ensemble model groups (mean Z-score = 189.0). HPV set attributes affected model quality much less than diverse algorithms. The most important among set attributes were the minimal recentness (mean Z-score = 49.0) and information capacity (39.1), while the number of time periods was least important (mean Z-score = 11.7). The model group itself was over eight times less important (mean Z-score = 23.1).

**Table 1:** Relative importance (mean Z-score of the raw regression-type RF importance) and significance ≤ 0.01 of historical proximity variables (HPV) set attributes according to the Boruta meta-model of the BIOMOD2 model true skill statistics (TSS) values.

HPV set attributes	Mean Z-score	Significance
Model algorithm	189.0	yes
Minimal recentness	49.0	yes
Information capacity	39.1	yes
Single/ensemble model	23.0	yes
Number of time periods	11.7	yes

## Discussion and conclusions

### Invasive species distribution models quality with and without an addition of historical proximity variables

HPV addition can be considered a serious improvement for simple iSDM models. An inclusion of HPVs strongly increased average single model quality, from weak (TSS = 0.38) to at least good (TSS = 0.6). The increase was observed also in the ensemble model group, where models with HPVs were better than No HPV ensemble SDMs (TSS = 0.82 vs. 0.77). The increase in the quality is quite substantial in relation to the other published giant hogweed models with a statistical approach to SAC [34,36]. However, the Boruta meta-model revealed that the model algorithm inside the single and ensemble algorithm groups was the most important determinant of the model quality, much more than single vs. ensemble distinction alone. We expected this, keeping in mind the huge differences in predictive ability between very simple (e.g. SRE) and sophisticated (e.g. RF or GBM) model algorithms [20]. It is remarkable that no matter what the algorithm was, an addition of any HPV set has increased the TSS of the model, which demonstrates promising applicability of the method.

**Fig 8:**
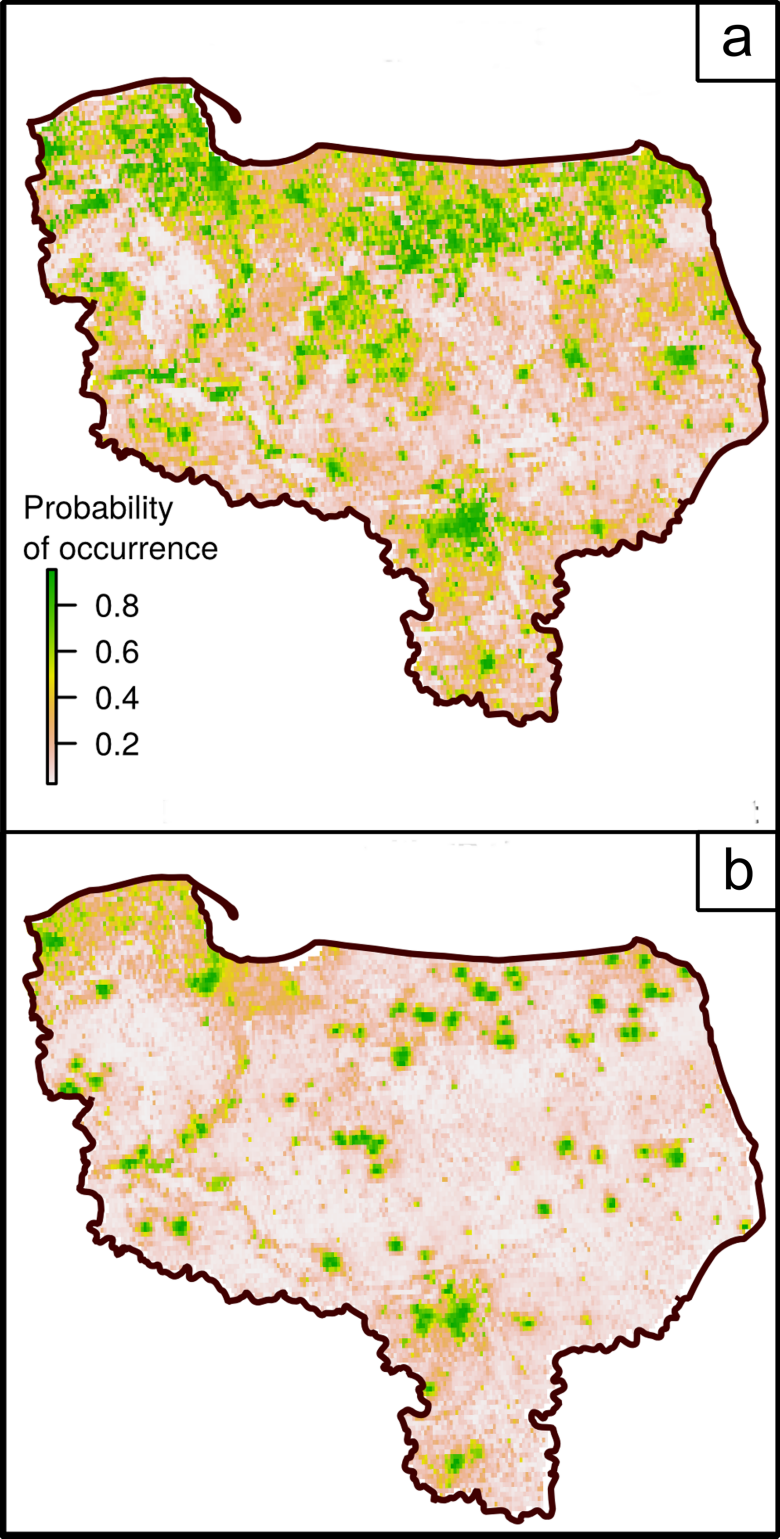
**Projection maps of probability of giant hogweed occurrence, using (a) no historical proximity variables and (b) with historical proximity variables of 5 years recentness of ‘Count’ and ‘Distance’ data applied in BIOMOD2 models.** Both maps are current weighted mean ensemble model projections of giant hogweed distribution in 2012.

The increase in model quality caused by the addition of biologically significant HPV variables is clearly visible in the model projections. The No HPV model does not manage to discern areas that are preferred by the species but inaccessible through usual dispersal (Fig 8A). In contrast to it, good HPVs model were excellent at limiting the probability of the species occurring strictly to the regions where the species was found (Fig 8B).

### Properties of historical proximity variables sets

The best variants were those with most recent HPVs and those with high information capacity (i.e. the distance to the recent sites), while those with low information capacity (such as the number of time periods included) were much less important. The Boruta meta-model confirmed that the most important determinants of model quality among HPV set attributes were minimal recentness and information capacity, which were about four times more important than the number of time periods included. The average quality of models decreases with increasing minimal recentness of the distance map, i.e. the length of the time span between the response data and the historical distribution (Fig 8B). This seems to confirm that the spatial dependence on the previous distribution weakens steadily with time in the analyzed giant hogweed invasion case.

The highest importance of the ‘Distance’ HPVs seems to also be an effect of the prevailing role of species expansion during the phase of *space infilling*. The existence of the site depends also on its persistence, but in our case, the minimal time span of HPV variables (5–20 yrs) was smaller than the minimal durability of giant hogweed populations (>50 yrs, [60]). In the case of more fugitive, early successional invasive species, the estimated effect of the mere Presence of previously existing sites or their Count may be much more important HPVs than in the giant hogweed case, due to the greater importance of the persistence of earlier sites rather than the creation of new sites only.

The inclusion of the ‘Distance’ HPV in the iSDM models should be a solution to the problem of propagule pressure confusion, i.e. the fact that the availability of propagules may be easily confounded with the plethora of populational and habitat factors [5,61,62]. The direct analysis of the distance HPV representing the spatially dependent probability of species migration together with many other potential invasion determinants should allow for much more successful estimation of their relative importance. It cannot replace individual or population-level experiments but can greatly help in initial understanding of the process and the setting of experiments.

Thus the case of giant hogweed invasion in the stage of *space infilling* seems to be well-suited for an application of HPV-based iSDM models. It may be an effect of the relative domination of short-distance dispersal of minimally winged large giant hogweed seeds, most of which land within a few meters of maternal plants [63–65]. Only a small amount of them travel far away, accidentally brought by wind, water flow or transportation [35,66]. Deliberate seed transport by humans was negligible in this area during the modelled period. In effect, most of the new, distant sites developed mostly within a few kilometers of the earlier sites. The suitability of the HPV approach implemented in this study may also result from the proper spatial resolution of the analysis. The raster cell size (1×1 km) was finely-grained enough to detect changes in site distribution during the time-span years. When the minimal recentness of HPVs reflects a longer time period, a larger site distribution would be detectable, and lower spatial resolution (larger cells) would be appropriate.

### Historical proximity variables of invasive species distribution models as a quasi semi-mechanistic approach

The HPV iSDMs cannot be considered a *semi-mechanistic approach*, because they do not include a typical mechanical model subsystem. However, the parameter that is estimated during calibration of the models is the *de facto* probability of the survival of an older giant hogweed site or emergence of a new one as a function of the properties of habitat (which is similar to classic SDMs and statistical approaches) and of the spatial properties of earlier species distribution (which is similar to a mechanical approach). Like the earlier semi-mechanistic approaches, (e.g. by Rouget and Richardson [28]), HPV iSDMs use SDM’s built-in mechanisms of calibration of spatially dependent parameters. In the case of our models it can be called a site dispersal curve; however, it is an analogue of the *inferring process from pattern* idea–but implemented on a larger spatial and biological scale. As the direct biological processes of invasion occur on the level of individuals, our models do not simulate basic biological mechanism as in a typical mechanical approach, therefore calling them *quasi semi-mechanistic* is appropriate.

The use of SDMs like those computed by the BIOMOD2 R package [25] allows also for obtaining a desirable measure of uncertainty, which is of high importance both for an understanding of the phenomena and for potential practical applications [3,21,67].

### Possible applications

**Fig 9:**
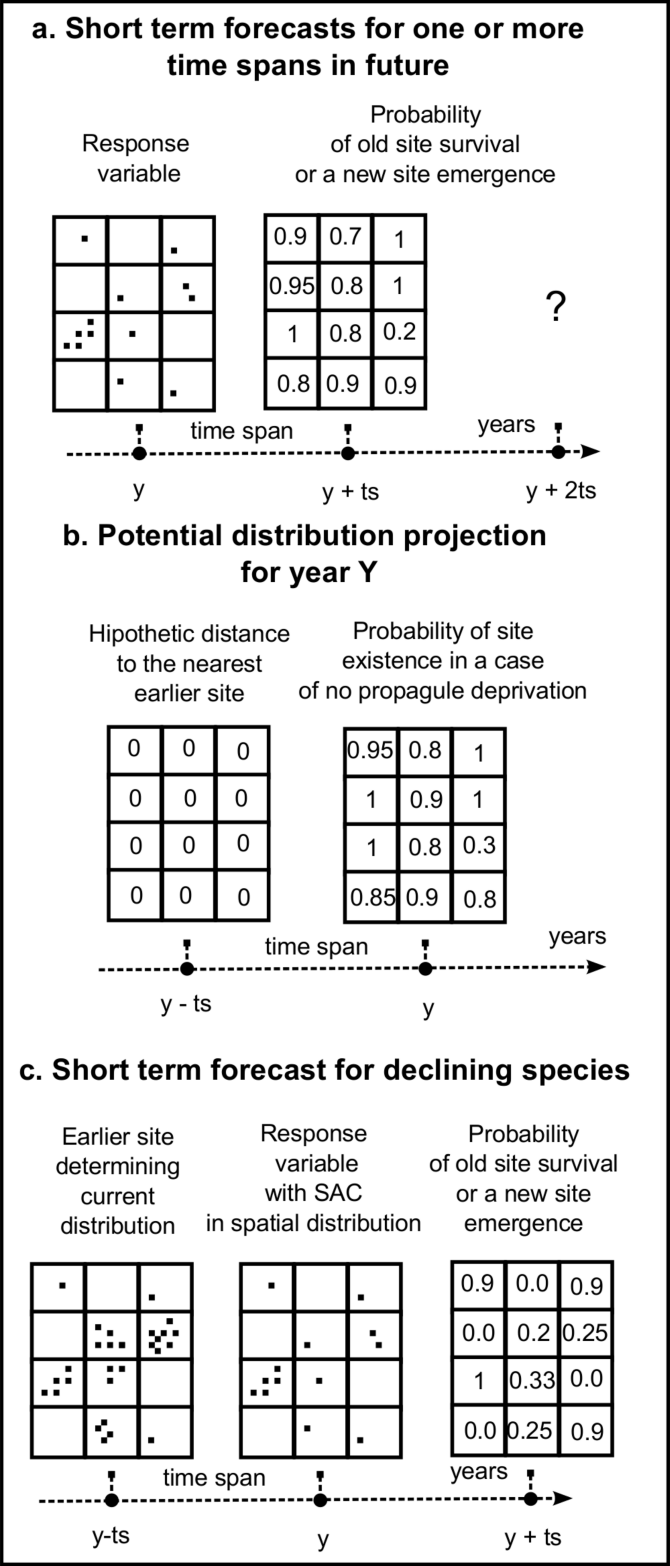
Possible applications of historical proximity variables (HPV) in quasi semi-mechanistic species distribution models (SDMs). (a) The inclusion and estimation of the explicit spatio-temporal relationships between the current and the earlier distribution of the modelled species enables one to make a forecast using the distance from the current distribution as one of the predictors. (b) The substitution of the distance to the nearest earlier site with low or zero values makes possible computing the equivalent of the potential range for invasive species. (c) The same idea should be applicable to the modelling of the declining species, where the distance and the number of the previous sites should ameliorate the estimation of the current distribution.

**Fig 10:**
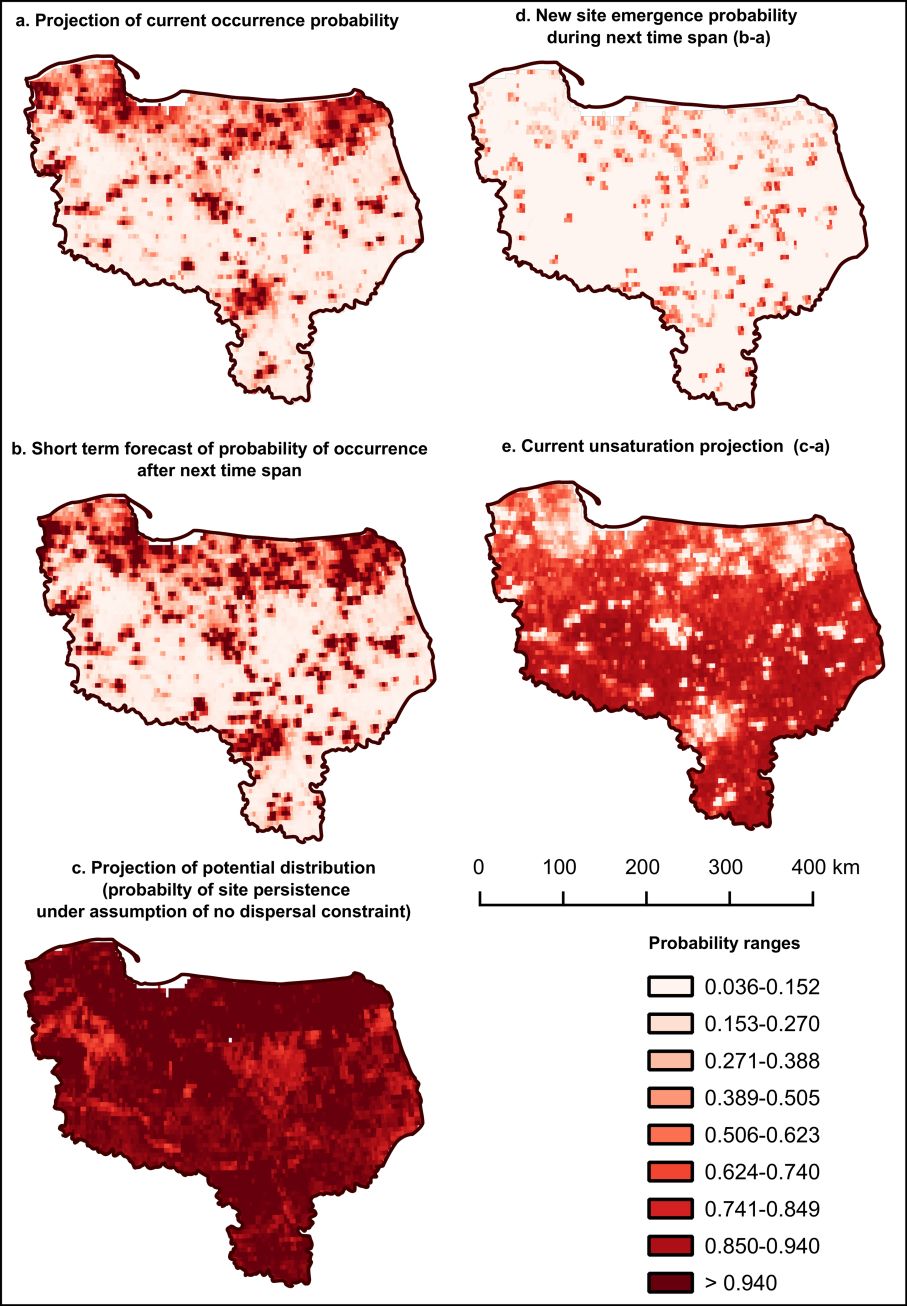
Projections of current, forecasted and potential distribution maps and their derivative products, produced using historical proximity variables (HPV) for the giant hogweed data from 2012, with distance to the nearest site 2 years earlier as an HPV variable [45]. Maps a-c are weighted mean projections of ensemble models with 3 algorithms (GBM, RF, CTA), 3 sets of 1500PA, 3 repetitions. ROC = 0.971. BIOMOD2 3.1.25, R 3.0.2.

The advantage of HPV iSDM models over the earlier semi-mechanistic approach lies in the explicit inclusion of time in the model. As there is a given time span between the response data and the historical proximity variables, the realistic rate of spread is estimated. Upon doing so, it can be also used to create a short-term forecast (Fig 9A). In such a case, the distance to the earlier sites can be substituted with the distance to the present ones, and instead of the projection of the current distribution (Fig 10A) one can obtain the forecast for the near future (present + time span, (Fig 10B)). It is even possible to iterate such a procedure, keeping in mind that spatial and temporal accordance between the response data and predictors is a requirement for proper logical inference in any SDM [3,19,20]. Thus in the case of sequential computing of the HPV of iSDMs–where the next iteration takes HPV values from its precedent–the predictor set should be amended in order to support the actuality and accuracy of the predictor set.

One of the ‘Holy Grails’ of iSDMs is the potential distribution management of invasive species. The obstacles to invasion originate from many successive barriers that must be surpassed during a successful process [68]. In the initial phases of an invasion process (*immigration*), every invasive species occurs in a small number of occurrences, which usually does not represent its true physiological niche. Models calibrated in this phase may underestimate the species’ future or potential distribution [7,8]. In later phases (*space infilling*), simple iSDMs lack the capability of separating habitat or climate constraints from the propagule deprivation. HPV iSDMs seem to be an effective solution to this problem in the regional spatial scale. They are developed on the base of the typical predictors describing habitat, climate, land use and cover, etc., together with HPVs representing various intrapopulation phenomena (e.g. population persistence, migration).

Successful construction of high-quality models makes possible the creation of projections or forecasts for different scenarios, including those with artificially set HPV values, e.g. in a case where the distance to the nearest previously existing site is very small. The model projection for this scenario can be interpreted as a probability of the site’s existence after the time equal to the minimal recentness of HPV used or a probability of the final space infilling (Fig 9B, Fig 10C). There is even a possibility of computing other maps exploring differences in the probability values of the abovementioned projections. Such maps might include a forecast of new site emergence (a projection of the probability forecasted for the near future minus the probability for the current occurrence, Fig 10D) or an unsaturation map (the probability of potential occurrence minus the probability for the current occurrence, Fig 10E).

The concept of HPV can be extended to other species groups that break assumptions of equal probability, e.g. rare or declining species like fen meadow species, whose occurrence probability depends on the distribution of their earlier sites (Fig 9C). The process of local extinctions may be more related to change of historic land use regimes, i.e. changes in suitable habitat availability. From the spatial and statistical point of view, present occurrences have been either “recruited” from former ones or settled in new places. In both cases (survival and colonization), clear spatio-temporal dependence of the distribution in year Y on the past distribution in year Y minus time span should exist and should be detectable by the HPV models. Thus HPV-based quasi semi-mechanistic SDMs are worth testing in such cases, as they may be both the simplest and most realistic tool for understanding and prediction available to ecologists and nature managers.

## Supporting information

S1 Table The list of worldgrids.org layers used as environmental predictors in the analyses. Detailed metadata can be found at http://worldgrids.org/doku.php/wiki:layers.
(XLS)

## Abbreviations

HPV: historical proximity values
SAC: spatial autocorrelation
SDM: species distribution model
iSDM: invasive species distribution model
TSS: true skill statistic
